# Tuberculosis as a primary cause of oesophageal stricture: a case report

**DOI:** 10.1186/s13019-018-0743-4

**Published:** 2018-06-05

**Authors:** Ronald Mbiine, Ronald Kabuye, Herve Monka Lekuya, William Manyillirah

**Affiliations:** 1 Department of Surgery, Makerere University, Kampala, Uganda; 2 Uganda Heart Institute, Kampala, Uganda

**Keywords:** Tuberculous oesophagitis, Oesophageal stricture, TB stricture

## Abstract

**Background:**

Tuberculous (TB) oesophagitis is a rare manifestation of dysphagia occurring in 0.3% of all gastro-intestinal tract TB infections as well as 0.15% of all cases of dysphagia and often is misdiagnosed. This report presents a rare manifestation of TB as a cause of oesophageal stricture.

**Case presentation:**

We describe a rare presentation of a patient with grade IV dysphagia due to an oesophageal stricture. Oesophagoscopy revealed a pinhole stricture with evidence of high grade dysplasia on histology. Subsequently an Ivor-Lewis oesophagectomy was performed and histology revealed evidence of active oesophageal tuberculosis. The patient had an uneventful recovery and completed anti-TB medication.

**Conclusions:**

Oesophageal TB is a rare but curable cause of dysphagia. It may mimic cancer of the oesophagus and it is usually missed as a possible cause of oesophageal strictures. There needs to be an increased index of suspicion among patients with dysphagia in TB endemic regions.

## Background

Tuberculous (TB) oesophagitis is a rare manifestation of dysphagia occurring in 0.3% of all gastro-intestinal tract TB infections as well as 0.15% of all cases of dysphagia and often is misdiagnosed [1]. This report presents a rare manifestation of TB as a cause of oesophageal stricture. Due to the rarity of this disease, often there may be delays in diagnosis which may as a result be associated with complications. Occasionally diagnostic challenges may also be encountered however there should be an increased index of suspicion in endemic populations.

The clinical presentation, diagnosis and treatment are discussed.

## Case presentation

### Patient information

We report on a 36-year old house-wife who presented to our hospital with a one-and-half year history of progressive dysphagia, first to solids and later to liquids. At the time of presentation she had Grade IV dysphagia. This was associated with progressive weight loss and retrosternal pain. She had no history of a chronic cough in the 1 year period and also had no history of treatment for TB. There was no family history of oesophageal diseases such as cancer.

### Clinical findings

On physical examination, she was in fair general condition with moderate wasting, she was not anaemic and neither was she jaundiced.

Overall, the systemic exam was unremarkable with no clinically significant findings in all systems.

### Diagnostic assesment

Initial blood work up included complete blood count, liver function, renal function and serum electrolytes which were all normal. She was sero-negative for Human Immunodeficiency Virus (HIV).

**Fig. 1:**
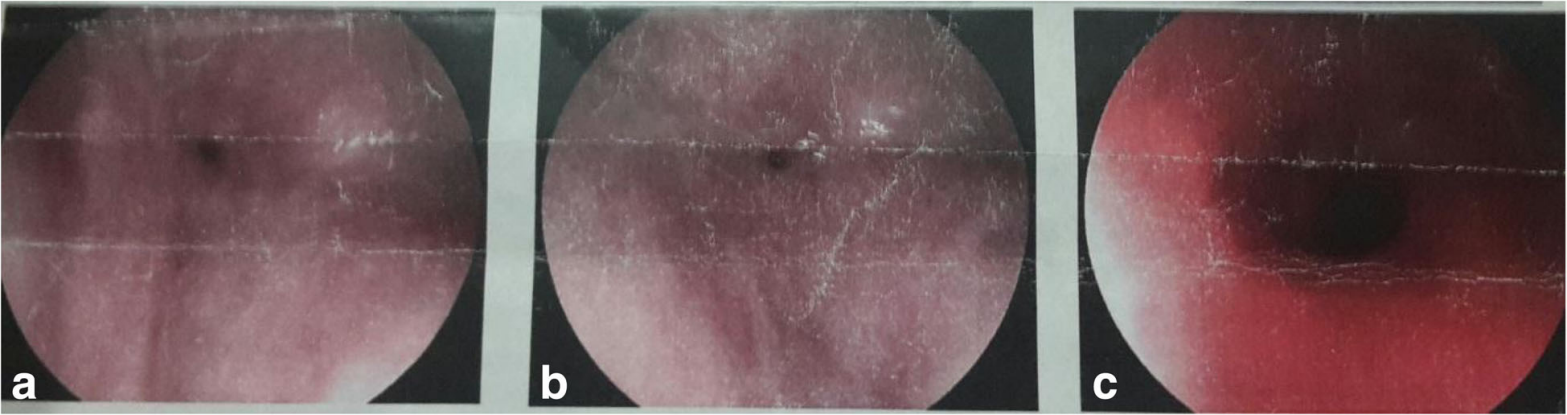
Upper GI endoscopy showing a pin-hole stricture with minimal mucosal inflammation at the proximal opening of the stricture. Legend: Fig. 1 a and b show a pin hole stricture of the oesophagus at 30 cm from the upper incisor teeth. Figure 1 c shows a patent oesophagus proximal to the stricture

Imaging investigations that were done included a barium swallow which revealed a 5 cm long mid-to-distal third stricture of the oesophagus. Following that, an upper gastrointestinal endoscopy was done which revealed a tight stricture at 30 cm with a pin-hole opening, minimal mucosal inflammation above the stricture with no ulceration or obvious mass. The scope could not be advanced beyond the narrowing (Fig. 1). The initial histology taken off at the time of endoscopy showed a high grade dysplasia (TIS). However, due to a delay in undertaking an oesophageal resection, a subsequent second endoscopy was done with repeat histology revealing a chronic inflammatory process. A staging chest and abdominal CT scan done showed an enhancing and markedly thickened oesophageal wall in the area of the stricture, but no abnormally enlarged intra-thoracic and intra-abdominal lymph nodes nor distant metastases were seen.

At that point basing on the findings of the initial histological findings of a high grade dysplasia, with no evidence of local or distant invasion, the patient was treated worked up and prepared for oesophagectomy.

#### Therapeutic intervention

**Fig. 2:**
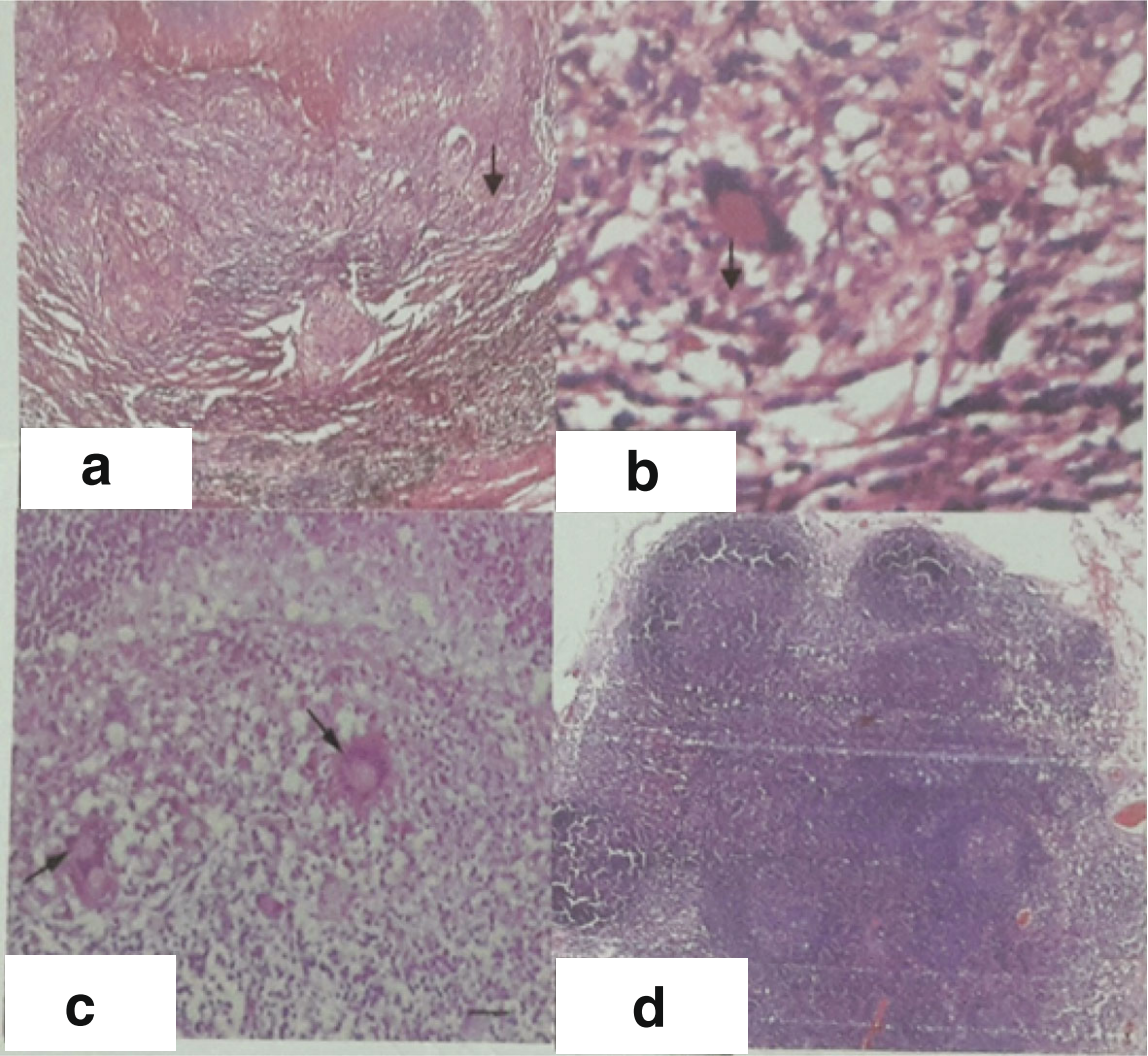
Histology of the surgical specimens. Legend: In Fig. 2 a and Fig. 2 b, histology of subcarinal lymph nodes shows well formed epitheliod granulomas and Langerhans giant cells (arrows) and caseous necrosis; Fig. 2 c stricture- shows normal stratified squamous epithelium but with areas of well formed epitheliod granulomas and Langerhans giant cells (arrows) and caseous necrosis; Fig. 2 d gastric/celiac lymph nodes- show marked reactive follicular hyperplasia

**Fig. 3:**
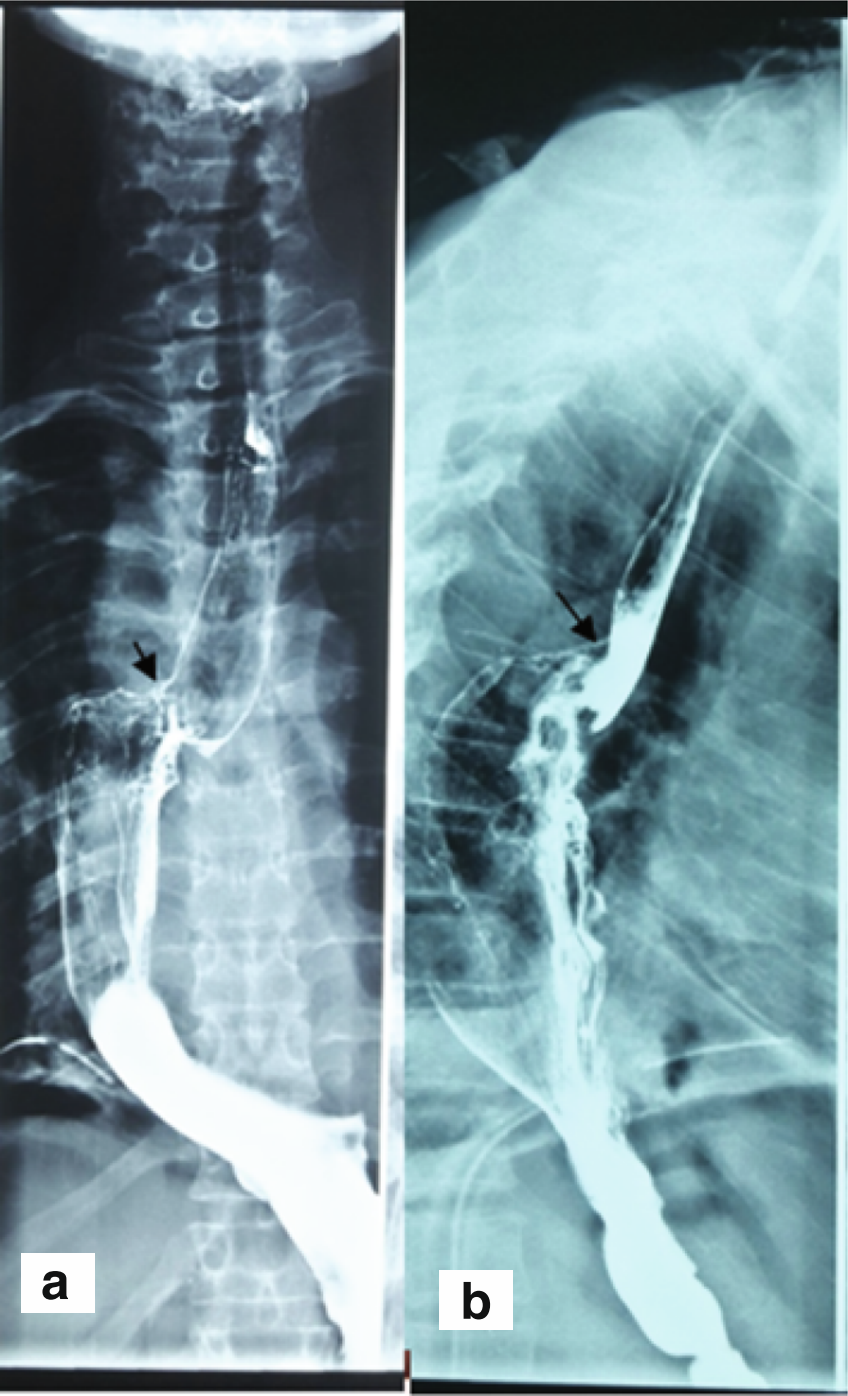
Day 7 post-operative barium swallow showing no anastomotic leak and no stenosis. Legend Fig. 3 a and b are anteroposterior and lateral views showing an intact anastomotic site as indicated by the black arrows

Initially, a temporary feeding gastrostomy was placed for nutritional rehabilitation and full optimisation for surgery. Due to the suspicion of an oesophageal carcinoma and the grade IV dysphagia, the patient was worked up for oesophagectomy. We performed an Ivor-Lewis oesophagectomy (upper midline laparotomy plus right thoracotomy with two-stage systematic lymph node dissection) with pyloplasty and a feeding jejunostomy. The resected oesophagus along with the dissected lymph nodes were sent for histology. The patient was extubated on table and transferred to the ICU where she spent 2 days. The results of the histology revealed active TB disease both at the stricture site and in the station 7 (sub-carinal) group of lymphnodes (Fig. 2). A barium swallow done on day 7 post surgery showed normal propulsion of barium, no stenosis and no anastomotic leak (Fig. 3).

The patient was started on anti-TB medications and was discharged on day 10 post surgery without any complications.

#### Follow up and outcomes

The patient was subsequently reviewed on numerous visits in the outpatient clinic. By the end of the first week following discharge, she had complete healing of the surgical incisions without any wound infection. She was continuously reviewed to ensure she didnot develop any short term and long term post surgical complications. Surgical OPD reviews were conducted first weekly for a month and then monthly for 6 months, TB treatment reviews were also done at the time of review of the surgical aspects. She took the anti-TB’s for 6 months and was reviewed monthly for treatment adherence, development of side effects. She adhered to her treatment and didnot develop any TB treatment complication. She had a complete recovery after the 6 months and has progressively resumed her daily activities. The follow up duration didnot exceed the 6 months following TB treatment and as a result the article was authored before follow up for any long term complications could be assessed.

## Discussions and conclusions

Oesophageal stricture is a very rare manifestation of extra-pulmonary tuberculosis globally. Despite the high prevalence of TB in sub-Saharan Africa, this is the first time oesophageal TB stricture is being documented in our population. The overall prevalence of TB oesophagus is less than 0.3% of all forms of gastrointestinal TB [1]. This has been attributed to the rapid clearance of oesophageal contents during swallowing. It has also shown to be present in up to 0.5% of the patients that present with dysphagia [2]. TB oesophagus may occur either as primary TB disease without evidence of pulmonary TB which has been doubted to exist. The other cases occur as either direct extension of mediastinal TB often presenting with complications such as trachea-oesophageal fistula. In our patient, a female, 36 years old, post oesophagectomy histology revealed active TB disease in the presence of an oesophageal stricture. The patient did not have evidence of active pulmonary TB. This thus could point to the possibility of primary TB oesophagitis without underlying lung disease. The presence and the nature of active mediastinal nodal disease in this patient implies either spread from the oesophagus to the mediastinal nodes but not vice versa.

The aetiology has been demonstrated to be mycobacterium tuberculosis however the mode of infection cannot be clearly elucidated. Primary inoculation following ingestion of infected material is the mode of infection for primary disease while direct extension from infected mediastinal structures has been attributed to the secondary type of infection [3]. Typically infection takes on similar pattern usually resulting in chronic granulomatous inflammation with giant cells and granuloma formation with areas of caseous necrosis. This usually is associated with ulceration of the mucosa which maybe symptomatic and often picked at endoscopy. In other cases, healing occurs with fibrosis with resultant development of the oesophageal stricture. Erosion into the trachea-bronchial tree with development of trachea-esophageal fistula or perforation into the mediastinum may also occur [4]. The most affected segment of the oesophagus is the middle third [5] owing to its proximity to the tracheobronchial tree and mediastinal nodes. However our case presented with a mid-distal third stricture and also concurs with a similar case which occurred in the distal third as well [4]. This is important as it questions the purported possibility of direct TB extension from the tracheobronchial tree or hillar lymph nodes.

In 90% of the patients, dysphagia is the commonest presentation [6], while other patients present with odynophagia, retrosternal pain and in some cases bleeding. Some cases will present with complications of tracheobronchial fistula and perforation [4]. Constitutional symptoms are often present but may not be ascribed to TB oeophagitis. Weight loss for example may be attributed to the malnutrition due to inadequate intake. Otherwise presence of weight loss, drenching night sweats following evening fevers have all been reported to be present [6]. However, in retrospective review of her history, over a period of 2 years she had had evening fevers, occasional night sweats, and household contact with a pulmonary TB patient who had, at the time of her surgery, completed an 8-month course of TB treatment.

The presence of active pulmonary or other extra-pulmonary TB disease need to be sought and history of chronic cough needs to be explored. Our patient did not have any chronic cough but had reported prior history of evening fevers and night sweats associated with weight loss. The diagnosis of oesophageal TB is usually made on oesophagoscopy and biopsy, where histology shows epithelioid granulomas with Langhans cells, central necrosis and acid-fast bacilli [4, 7, 8]. In our case, prior histology revealed high grade dysplasia and subsequently inflammatory tissue without conclusion of TB. The inability to advance the scope into the lumen of the stricture prevented us from taking biopsies from the culprit mucosa. In other cases, the histological confirmation is made following surgery with resection of the oesophageal stricture [9, 10]. Once TB diagnosis is made, chest x-ray, abdominal ultra sound scan are done to rule out presence of TB in other areas. The chest x-ray may be normal in the majority of the patients [5] while upper gastrointestinal-contrast studies often show stricture that may mimic a malignant stricture. Strictures maybe due to luminal, intramural or extrinsic compression depending on the form of TB involvement [5]. Contrast leakage into the mediastinum or into the tracheobronchial tree may also be demonstrated in the presence of fistulation.

Thoracic contrast enhanced CT scan may also delineate presence of the oesophageal lesion or presence of extrinsic mediastinal nodes compressing the oesophagus. The oesophageal thickness is also assessed. In our patient, chest CT scan showed a markedly thickened and enhancing oesophageal wall at the stricture level, but no obvious extrinsic masses or lymphadenopathy. However, at operation, pathological mediastinal lymphnodes were found especially at station 7 (sub-carinal). Typically once the diagnosis is made, the patient is started on anti-TB medication and response has been described as sufficient [2, 4]. However the presence of complications warrants surgical intervention [5, 10] and this maybe in the form of treatment of the stricture following principles of stricture management. In our case, an Ivor-Lewis oesophagectomy was performed because of total oesophageal occlusion with suspicion of malignancy. .

The patient had an un-eventful recovery in the immediate postoperative period and was discharged on the 10th post-operative day. She was enrolled on anti-TB medication and was followed up post TB treatment with a successful recovery.

## Conclusion

Oesophageal TB is a rare but curable cause of dysphagia. It may mimic cancer of the oesophagus and it is usually missed as a possible cause of oesophageal strictures. There needs to be an increased index of suspicion among patients with dysphagia in TB endemic regions. In uncomplicated cases, anti-tubercular treatment is adequate for cure and resolution of dysphagia while once complications occur, surgical intervention may be necessary. Overall the outcome is excellent once proper diagnosis is made.

## Abbreviations

CT: Computed tomography
HIV: Human immunodeficiency virus
MDR TB: Multi drug resistant tuberculosis
TB: Tuberculosis
TIS: Tumour in situ
